# Resveratrol Protects against *Helicobacter pylori*-Associated Gastritis by Combating Oxidative Stress

**DOI:** 10.3390/ijms161126061

**Published:** 2015-11-20

**Authors:** Xiaolin Zhang, Anmin Jiang, Banghua Qi, Zhongyou Ma, Youyi Xiong, Jinfeng Dou, Jianfei Wang

**Affiliations:** 1 College of Food and Drug, University of Anhui Science and Technology, Bengbu 233100, China; qibh@ahstu.edu.cn (B.Q.); mazy@ahstu.edu.cn (Z.M.); xyytc1@163.com (Y.X.); doujf@ahstu.edu.cn (J.D.); 2 The School of Life Science, University of Science and Technology of China, Hefei 230032, China; SA12226011@mail.ustc.edu.cn; 3 The Ministry of Agriculture Key Laboratory of Microbial Organic Fertilizer, Bengbu 233030, China

**Keywords:** resveratrol, oxidative stress, gastritis, iNOS, IL-8, Nrf2, HO-1

## Abstract

*Helicobacter pylori* (*H. pylori*)-induced oxidative stress has been shown to play a very important role in the inflammation of the gastric mucosa and increases the risk of developing gastric cancer. Resveratrol has many biological functions and activities, including antioxidant and anti-inflammatory effect. The purpose of this study was to probe whether resveratrol inhibits *H. pylori*-induced gastric inflammation and to elucidate the underlying mechanisms of any effect in mice. A mouse model of *H. pylori* infection was established via oral inoculation with *H. pylori*. After one week, mice were administered resveratrol (100 mg/kg body weight/day) orally for six weeks. The mRNA and protein levels of iNOS and IL-8 were assessed using RT-PCR, Western blot and ELISA. The expression levels of IκBα and phosphorylated IκBα (which embodies the level and activation of NF-κB), Heme Oxygenase-1 (HO-1; a potent antioxidant enzyme) and nuclear factor-erythroid 2 related factor 2 (Nrf2) were determined using Western blot, and lipid peroxide (LPO) level and myeloperoxidase (MPO) activity were examined using an MPO colorimetric activity assay, thiobarbituric acid reaction, and histological-grade using HE staining of the gastric mucosa. The results showed that resveratrol improved the histological infiltration score and decreased LPO level and MPO activity in the gastric mucosa. Resveratrol down-regulated the *H. pylori*-induced mRNA transcription and protein expression levels of IL-8 and iNOS, suppressed *H. pylori*-induced phosphorylation of IκBα, and increased the levels of HO-1 and Nrf2. In conclusion, resveratrol treatment exerted significant effects against oxidative stress and inflammation in *H. pylori*-infected mucosa through the suppression of IL-8, iNOS, and NF-κB, and moreover through the activation of the Nrf2/HO-1 pathway.

## 1. Introduction

*Helicobacter pylori* (*H. pylori*) infection causes gastric and duodenal inflammation which leads to ulceration and gastric carcinoma [1,2]. The previous report has shown that *H. pylori* infection induces pathologic changes of the congestive and edema of the gastric surface epithelium [3]. *H. pylori*-associated gastritis is an inflammatory response associated with mucosal injury, hypochlorhydria and neutrophil infiltration. A characteristic event of *H. pylori*-associated gastritis lies in the production of large amounts of reactive oxygen species (ROS) that induce the expression of inflammatory genes [4]. However, *H. pylori* is a non-tissue cell invasive bacteria; as such, it induces the expression of inflammatory genes through a signaling cascade pathway. Several mediators play important roles in this process. Chemokines are a family of pro-inflammatory mediators that play considerable roles in the expression of inflammatory proteins. The reports have revealed that chemokine interleukin-8 (IL-8) are overexpressed in gastric tissue samples of patients suffering from *H. pylori*-caused gastritis. *H. pylori*-caused gastric mucosal inflammation and damage are mediated by the overproduction of nitric oxide, a reactive nitrogen species that can be inducibly generated by inducible nitric oxide synthase (iNOS). Studies have shown that iNOS is an inducible inflammatory enzyme and an important pathogenic factor in *H. pylori*-caused gastritis [5]. Constant overproduction of nitric oxide may lead to DNA and tissue damage, which could increase the risk of developing cancer [6]. The expression of the inflammatory mediator IL-8 and the enzyme iNOS is regulated by NF-κB [7,8,9,10,11]. NF-κB is composed of either a p50 homodimer or a p50/p65 heterodimer [12] and binds the inhibitory protein IκBα in the cytoplasm under normal physiological conditions. The rapid proteasomal degradation of IκBα can be triggered upon sensing extracellular stimuli, and it translocates into the nucleus and bind to DNA regulatory sites of target genes involving the pro-inflammatory mediator IL-8 and the enzyme iNOS [13]. It has been shown that if the NF-κB signal transduction pathway is activated, it would be an important event linked to tumorigenesis [14]. NF-κB can be activated by ROS, as elicited by *H. pylori* in neutrophils. Upon *H. pylori* infection of the stomach, ROS can damage gastric mucosal cells and cause the peroxidation of membrane lipids, thereby increasing the level of lipid peroxide (LPO) in damaged tissues [15]. In addition, myeloperoxidase (MPO) activity is a biomarker of neutrophil infiltration due to its higher expression in neutrophils than in other cells [16]. Therefore, high levels of LPO and an increase in MPO activity are thought to reflect the degrees of oxidative damage and inflammatory response, respectively, in cells. Interestingly, cells can survive the damage caused by anti-chronic oxidative stress by enhancing the activity of anti-oxidant enzymes. Previous studies have demonstrated that heme oxygenase-1 (HO-1) is an inducible antioxidant enzyme that exhibits a wide range of adaptive responses and represents a therapeutic target in the fight against oxidative stress and gastrointestinal diseases [17,18]. Enhancing the level of this enzyme may be a therapeutic strategy for protecting against oxidative damage. Nuclear factor erythroid 2-related factor 2 (Nrf2) is an important transcriptional regulator of HO-1. Nrf2 is thought to act as a cellular sensor of oxidative stress and as a redox switch that turns on cellular signaling and induces expression of cytoprotective genes [19,20,21]. Therefore, Nrf2 is a key target of antioxidant enzymes to convert highly toxic and active ROS into less damaging and less reactive forms.

Oxidative stress, through the generation of reactive nitrogen and oxygen species, plays an important role in *H. pylori*-induced gastritis. Therefore, compounds with antioxidant or anti-inflammatory effects, particularly compounds derived from plants, for protecting against *H. pylori*-associated gastritis and reducing the incidence of gastric cancer are highly valuable. Resveratrol, which is found in several dietary plant sources including berries, nuts, peanuts, and grape skin, has been shown to have potent anti-inflammatory [22] and antioxidant [23] properties. Resveratrol can prevent or defer the occurrence and progression of various illnesses, including cardiovascular ailments, neurodegenerative diseases, ischemic injury, and viral infection, and can even extend the life span of diverse organisms and inhibit the growth of various cancer cells [24,25,26,27,28]. The primary goal of the present study is to investigate whether resveratrol has the ability to protect experimental animals against *H. pylori*-induced chronic gastric inflammation. Animal models of *H. pylori* infection were established by orally inoculating mice with clinical isolates of *H. pylori*; then, changes in the level of LPO, which responds to oxidative damage of the membrane, and in the activity of MPO, which acts as a biomarker of neutrophil infiltration, were determined. We also examined the expression levels of the pro-inflammatory mediator IL-8 and the enzyme iNOS; the phosphorylation level of IκBα, which reflects the activation of NF-κB; and levels of the antioxidant enzyme HO-1 and its transcriptional regulator Nrf2. Moreover, the number of viable *H. pylori* colonies and histological pathological scores were compared and analyzed in gastric biopsy samples between animals that were treated with resveratrol and those that were not. Our results demonstrated that resveratrol decreases *H. pylori*-induced gastric inflammation by suppressing the *H. pylori*-induced pro-inflammatory mediator IL-8 and the expression of iNOS while up-regulating HO-1 and Nrf2 expression in tissues affected by *H. pylori*-associated gastritis.

## 2. Results and Discussion

### 2.1. Effect of Resveratrol on the Colonization Number of H. pylori in the Stomach

**Figure 1:**
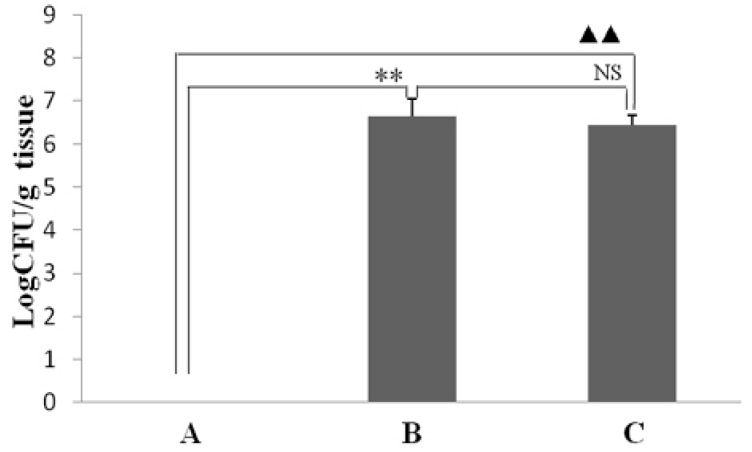
The effect of resveratrol on the colonization number of *H. pylori* in the gastric mucosa of mice. The colonization number of *H. pylori* was determined and represented as each gram gastric tissue that can generate colony forming units (CFU)/g. (A) Normal control group: animals were fed the control diet and not found colonization of *H. pylori*; (B) *H. pylori*-infected model group: animals were fed the control diet and found colonization of *H. pylori*; (C) *H. pylori*-infected mice treated with resveratrol group: animals were fed a diet supplemented with resveratrol and found colonization of *H. pylori*. ** *p* < 0.01 and ^▲▲^ *p* < 0.01 *versus* normal animals; NS: no significant differences *versus* model animals (*n* = 10).

To determine whether resveratrol influences the effect of *H. pylori* colonization in the gastric mucosa of infected mice, the existence of *H. pylori* in the stomachs was determined after six weeks of treatment with resveratrol. As seen in Figure 1, resveratrol treatment had no effect on colonization number of *H. pylori* in the stomach.

### 2.2. Effect of Resveratrol on H. pylori-Caused Gastric Tissue Inflammation

**Figure 2:**
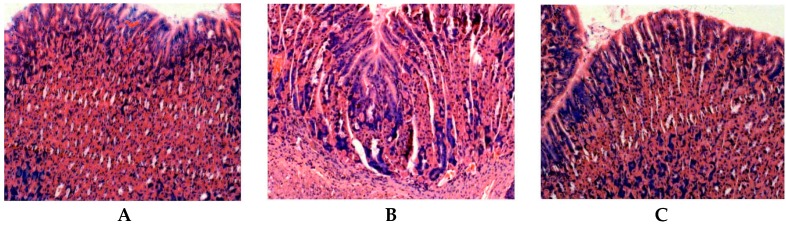
The observation of resveratrol on *H. pylori*-caused histological changes by histopathological analysis of the mouse stomach (HE stain, 200×) (**A**) Normal control animals: Epithelial cells are arranged in neat rows and there are very few inflammatory cells scattered in epithelial tissues. Scored 0.25 ± 0.03; (**B**) *H. pylori*-infected model animals: Heavy infiltration of granulocytes, lymphocytes and plasma cells in tissues. Scored 2.87 ± 0.46; (**C**) *H. pylori*-infected animals treated with resveratrol: Infiltration of only a few lymphocytes and inflammation in epithelial tissues. Scored 1.3 ± 0.18.

The animals infected with *H. pylori* developed severe gastritis after 6 weeks. As seen in Figure 2, the gastric mucosal regions that infected animals exhibited obvious neutrophil infiltration, tissue congestion and lymphoid follicle formation (Figure 2B) compared with those that uninfected normal animals (Figure 2A). The gastric mucosal tissue of resveratrol-treated animals exhibited obvious improvement of inflammatory cell infiltration (Figure 2C). The results of histological grading suggest that resveratrol antagonizes *H. pylori*-induced gastric inflammation and improves the histopathological grade of gastritis in mouse stomachs.

### 2.3. Effect of Resveratrol on H. pylori-Caused MPO Activity and LPO Level

**Figure 3:**
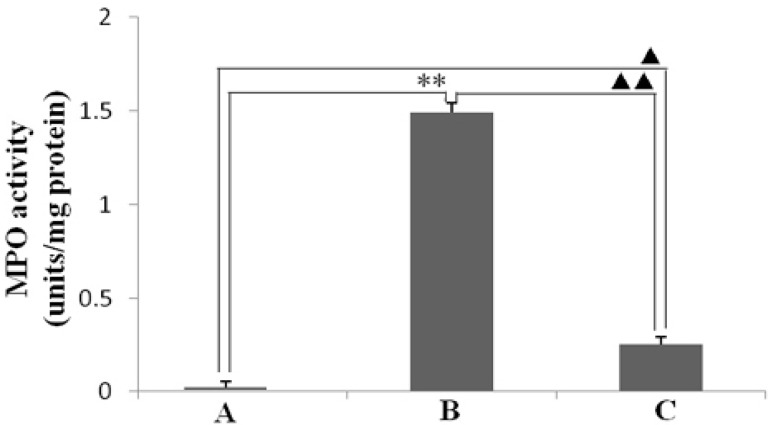
The effect of resveratrol on myeloperoxidase (MPO) activity (units/mg protein) in the gastric mucosal tissues of mice infected with *H. pylori*. (A) Normal control animals; (B) *H. pylori*-infected model animals without resveratrol treatment; and (C) *H. pylori*-infected animals with resveratrol treatment. ** *p* < 0.01 and ^▲^ *p* < 0.05 *versus* normal animals; ^▲▲^ *p* < 0.01 *versus* model animals (*n* = 10).

**Figure 4:**
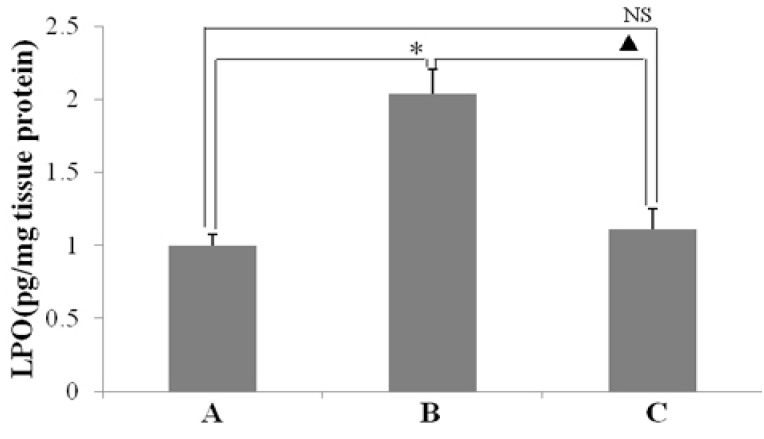
The effect of resveratrol on lipid peroxide (LPO) level (pg/mg protein) in the gastric mucosal tissues of mice infected by *H. pylori.* (A) Normal control animals; (B) *H. pylori*-infected model animals; and (C) *H. pylori*-infected animals treated with resveratrol. * *p* < 0.05 *versus* normal animals; ^▲^ *p* < 0.05 *versus* model animals; NS: no significant differences *versus* normal animals (*n* = 10).

As seen in Figure 3, *H. pylori* infection caused an enhancement of MPO activity in the gastric mucosa (Figure 3B). However, resveratrol inhibited increase of MPO activity in gastric mucosal tissues that infected with *H. pylori* (Figure 3C). Moreover, this result showed that the suppression of MPO activity by resveratrol was associated with a reduction of *H. pylori*-caused neutrophil infiltration in the gastric mucosa (Figure 2C). As shown in Figure 4, LPO level, which reflects the oxidative damage and degree index, was markedly higher in the tissues of animal gastric mucosa infected by *H. pylori* than in those of non-infected normal animals. This demonstrates that resveratrol treatment inhibits the increase of LPO level owing to *H. pylori*-induced in gastric mucosal tissue.

### 2.4. Effect of Resveratrol on H. pylori-Caused Expression of IL-8 and iNOS, HO-1 and Nrf2, Level of Phosphorylation of IκBa

**Figure 5:**
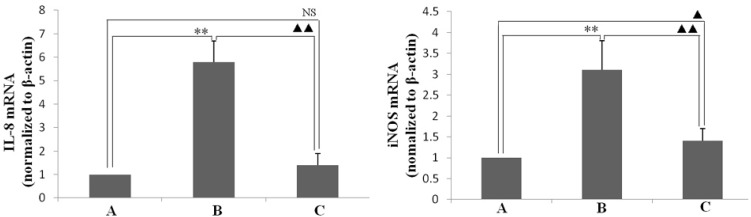
The effect of resveratrol on *H. pylori*-induced mRNA expression of interleukin-8 (IL-8) and inducible nitric oxide synthase (iNOS) in the gastric mucosal tissues of mice. Quantitative RT-PCR was performed based on reverse-transcribed RNA from gastric mucosal tissues. The levels of IL-8 and iNOS mRNA were normalized to the level of internal control β-actin. (A) Normal control animals; (B) *H. pylori*-infected model animals without resveratrol treatment; and (C) *H. pylori*-infected mice with resveratrol treatment. ** *p* < 0.01 *versus* normal animals; ^▲^ *p* < 0.05 *versus* normal animals; ^▲▲^ *p* < 0.01 *versus* model animals; NS: no significant differences *versus* normal animals (*n* = 10).

**Figure 6:**
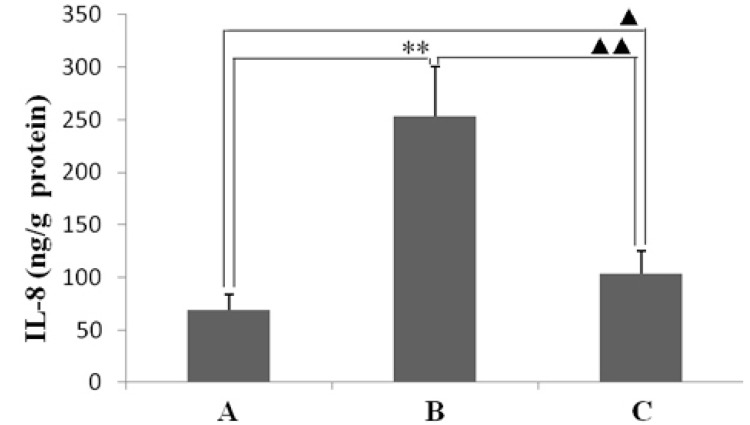
The effect of IL-8 protein levels in gastric mucosal tissues with resveratrol treatment, as measured by ELISA. (A) Normal control animals; (B) *H. pylori*-infected model animals without resveratrol treatment; and (C) *H. pylori*-infected animals with resveratrol treatment. ** *p* < 0.01 *versus* normal animals; ^▲▲^ *p* < 0.01 *versus* model animals; ^▲^ *p* < 0.05 *versus* normal animals (*n* = 10).

**Figure 7:**
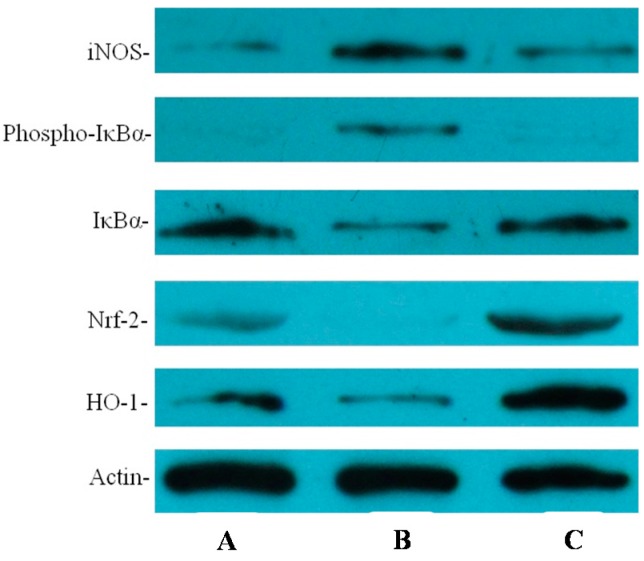
The effect of resveratrol on the protein levels of inducible nitric oxide synthase (iNOS), the phospho-specific form of IκBa, IκBa, Nrf2, and HO-1 in *H. pylori*-infected gastric mucosal tissues as determined by Western blotting. (A) Normal control animals; (B) *H. pylori*-infected model animals without resveratrol treatment; and (C) *H. pylori*-infected animals with resveratrol treatment.

IL-8 and iNOS are important inflammatory mediators of *H. pylori*-caused inflammation. Therefore, in this study, the levels of IL-8 and iNOS mRNA and protein in the animal gastric mucosal tissues that infected by *H. pylori* were determined. As shown in Figure 5, the expression levels of IL-8 and iNOS mRNA in gastric mucosal tissues of animals infected by *H. pylori* were higher than in ones of normal animals non-infected. However, *H. pylori*-induced expression levels of IL-8 and iNOS mRNA was obviously lower in the animals infected by *H. pylori* and treated with resveratrol than untreated animals. The increases in IL-8 and iNOS protein levels induced by *H. pylori*-infected were also ameliorated in *H. pylori*-infected animals treated with resveratrol, as detected by enzyme-linked immunosorbent assay or Western blotting, respectively (Figure 6 and Figure 7).

The inflammatory mediators of IL-8 and iNOS are modulated by NF-κB, which is an oxidant-sensitive transcription factor. The level of IκBα represents the activated status of NF-κB. As seen in Figure 7, the level of IκBα phosphorylated was higher in animals infected by *H. pylori* than in uninfected animals and was obviously lower in animals treated with resveratrol than in untreated animals. However, IκBα was lower in animals infected by *H. pylori* than in uninfected normal animals, moreover, resveratrol-treated animals reversed the IκBα protein level in gastric mucosal tissues infected by *H. pylori*. These results suggest that resveratrol treatment may inhibit the activation of NF-κB by suppressing the level of IκBα phosphorylated in the gastric mucosa of mice infected by *H. pylori*. 

Because HO-1 and Nrf2 have been shown to exert important roles in suppressing inflammation and protecting against oxidative stress, we assessed whether resveratrol affected HO-1 protein expression. As shown in Figure 7, *H. pylori*-infected animals treated with resveratrol induced greater expression of HO-1 proteins than did untreated animals. Several studies have demonstrated that Nrf2 is one of important transcription factors for promoting HO-1 expression [19,20,21]. We further examined whether resveratrol induces the expression of Nrf2 in *H. pylori-*infected animals. Our results demonstrated that the Nrf2 level was higher in animals infected by *H. pylori* and treated with resveratrol than in untreated controls. Moreover, the increase in Nrf2 was correlated with an increase in HO-1 expression (Figure 7).

In this study, the anti-inflammatory effect on resveratrol was investigated in mice using an *H. pylori*-caused gastric inflammation model. Resveratrol was found to exert a significant protective effect against *H. pylori*-caused gastritis. Our analysis using tissue pathology demonstrated that resveratrol lessens *H. pylori*-induced gastric injury (Figure 1). Previous studies showed that resveratrol was effective inhibitory effect for *H. pylori* growth or increased susceptibility to resveratrol of *H. pylori* strains *in vitro* [29,30,31]. In this study, the extent of *H. pylori* colonization of gastric mucosal tissue of animals infected by *H. pylori* and treated with resveratrol did not differ from that of untreated animals (Figure 2). One possible explanation is that resveratrol is poorly soluble, and the bacteria are found under mucosa where resveratrol does not penetrate. Another reason may be gastric emptying: the residence time of resveratrol in the stomach is short. Moreover, a characteristic event in *H. pylori*-associated gastritis is that the production of large amounts of ROS and reactive nitrogen species (RNS) likely leads to an imbalance between removing the production of ROS/RNS and antioxidant defenses, which is the main cause of chronic gastritis [4,32,33]. ROS and RNS can cause DNA damage and induce gastric carcinogenesis in gastric mucosa infected by *H. pylori* [34]. Excessive production of ROS/RNS causes irreversible damage and destruction to a variety of cellular components, including proteins, nucleic acids, and lipids [35,36]. ROS is an important factor for the lipid peroxidation of cell membrane. LPO is an important cause of cell membrane damage that results in the pathogenesis visible in many gastric diseases, including *H. pylori*-associated gastritis. Moreover, the LPO level and MPO activity in the gastric mucosal tissues of mice infected by *H. pylori* are increased compared to those of normal mice. However, resveratrol treatment inhibited MPO activity (Figure 3) and reduced LPO level (Figure 4) in the stomach. The ability of resveratrol to prevent the increase of LPO level induced by *H. pylori* infection could be associated with a reduction of MPO activity. LPO level is directly related to ROS production [37,38], and the principal source of ROS production is neutrophil activation by *H. pylori* infection [39,40]. This is consistent with the reduction of neutrophilic infiltration reported in previous observations of tissue pathology (Figure 2). Furthermore, MPO activity was directly related to the amounts of neutrophils, and could be used to quantitate inflammation [41]. ROS can activate oxidant-mediated transcription factors including NF-κB, which in turn induces the expression of the pro-inflammatory mediator IL-8 and enzyme iNOS. In this study, we found that resveratrol inhibits NF-κB activity by reducing IκBα phosphorylation, and decreases iNOS and IL-8 expression (Figure 5, Figure 6 and Figure 7). These results suggest that resveratrol has a protective effect against *H. pylori*-induced gastric inflammation. Moreover, inducible antioxidant enzymes have important protective effects against oxidative stress-related diseases and oxidative injury, including chronic gastritis. HO-1, one of the most important inducible antioxidant enzymes, exhibits a wide range of adaptive responses to oxidative stress. Therefore, the discovery of antioxidant substances that induce the activity of this enzyme is considered an important therapeutic strategy against oxidative damage [17,18]. Nrf2 is a very important transcription factor that induces HO-1 expression. It plays the role of cellular sensor of diverse oxidative stressors and represents an important stress response element for turning on the cellular signaling pathway active in the cellular redox state [19,20,21]. Therefore, Nrf2 is considered a key regulator and antioxidant enzyme inducer for protection against oxidative and inflammatory stress. In this study, we found that resveratrol up-regulates HO-1 and Nrf2 expression in *H. pylori*- infected gastric mucosa (Figure 7). In conclusion, resveratrol treatment exerted significant antioxidant and anti-inflammatory effects in *H. pylori*-infected mucosa by suppressing the expression IL-8 and iNOS, blocking the activation of NF-κB, and increasing the expression levels of Nrf2 and HO-1. In short, the resveratrol at a dose of 100 mg/kg body weight/day exerted a protective effect for *H. pylori*-induced gastritis in a mouse model of *H. pylori* infection. The results indicate that resveratrol at a dose of about 1 g/day was able to prevent from *H. pylori*-associated gastritis and reduce the incidence of gastric cancer for humans.

## 3. Experimental Section

### 3.1. Animals and Resveratrol

Six-week-old male Kunming mice were purchased from Changlinhe Pharmaceutic Co., Ltd., Feidong, China. The mice were housed in individual ventilation cages and fed a specific pathogen free (SPF) laboratory animal pellet diet and sterile water *ad libitum*. All experimental procedures were carried out according to Chinese legislation No. 8910M047 about the care and use of laboratory animals. The regulations were formulated and approved by the Institute for Experimental Animals Committee of Anhui Science and Technology University. Thiobarbituric acid (TBA) and hexadecyltrimethylam-monium bromide (HTAB) were obtained from Sangon Biotech Co. Ltd. (Shanghai, China). Resveratrol (contents > 98%) was purchased from Ciyuan Pharmaceutical Co., Xi’an, China and the contents were determined using analysis of high performance liquid chromatography.

### 3.2. Growth of H. pylori and Inoculation of Mice

Two *H. pylori* clinical strains were isolated from two patients with gastric cancer and gastric ulcer and were preserved in our laboratory at −80 °C in brain heart infusion (BHI) broth (Hope Biol-technology Co., Ltd., Qingdao, China) supplemented with 10% fetal bovine serum (FBS) (Sangon Biotech Co., Ltd.) and 20% glycerol. Bacteria were grown on BHI agar (Hope Biol-technology Co., Ltd.) supplemented with 7% defibrinated sheep blood (Jiulong Biological Products Co., Ltd., Zhenzhou, China), 10 μg/mL vancomycin, 5 μg/mL cefsulodin, 5 μg/mL polymyxin B, 5 μg/mL trimethoprim at 37 °C under microaerophilic conditions generated using an MGC Anaero Pack which can produce microaerophilic atmosphere in a sealed jar (Mistsubishi Gas Chemical Company, Inc., Tokyo, Japan). For bacterial amplification and number counts, *H. pylori* was cultured in BHI broth containing 10% FBS. Cultures were shaken in a microaerobic environment. Bacteria were collected and diluted to 1 × 10^8^ CFUs/mL according to the growth curve prior to the infection of each animal. Each mouse was challenged with a 0.3 mL (10^8^ CFUs/mL) dose of *H. pylori* on each of three consecutive days.

### 3.3. Experimental Design

Thirty experimental animals were randomized into three groups. Mice in Group A (*n* = 10) were normal controls not inoculated with *H. pylori*. Twenty mice were orally inoculated 10^8^ CFU *H. pylori* for three times to establish model of *H. pylori* infection. One week after the last *H. pylori* inoculation, mice were randomly divided into two groups. The mice in Group B (*n* = 10) were not given resveratrol; the mice in Group C (*n* = 10), the treatment group, were administered dietary resveratrol (100 mg/kg body weight/day) for six continuous weeks. Resveratrol was dissolved in absolute ethanol, they were evenly added mouse feed then freeze-dried under protected from light condition. The mice had free access food supplemented with resveratrol equivalent to 100 mg/kg body weight/day. The doses of resveratrol have been calculated based on dietary concentrations of resveratrol, food intakes, and body weights of animals at various time points of the study as previously reported [42]. At the end of the experiment, a dose of 3% pentobarbital sodium solution was used to euthanize each mouse. Each mouse stomach was longitudinally cut into three equal parts and each part contains antrum and body. The first portion of each biopsy specimen was collected for bacterial culture to confirm *H. pylori* colonization. The second portion of each stomach was fixed using formalin, and embedded using paraffin, and then cut into sections and stained with hematoxylin and eosin (HE) to analyze *H. pylori-*associated inflammation. The remainder of the stomach was used to determine LPO level; MPO activity; IL-8 and iNOS mRNA transcriptional levels; and protein expression levels of IL-8, iNOS, phospho-specific IκBα, IκBα, HO-1, and Nrf2.

### 3.4. Determination of H. pylori Colonization in Mouse Stomach

In each mouse stomach tissue sample, the *H. pylori* colonization was determined using a microbial culture method as described in our previous report [43]. Stomach tissues were fully homogenized in 1.5 mL BHI broth under sterile environment with a tissue homogenizer. The homogenate was 10-fold serially diluted with BHI broth and then plated on *H. pylori*-selective BHI agar plates added with above antibiotics and 7% defibrinated sheep blood as the methods described above. All the plates were cultured under microaerobic conditions using above methods for 3 days at 37 °C. The colonization number of *H. pylori* was determined by counting and calculated bacterial colonies on the each agar plate (at least in triplicate). Then the colonization number of bacteria per gram of mouse gastric tissue was calculated and expressed as the logarithm of colony forming units (CFUs)/g of per gram gastric biopsy sample.

### 3.5. Histological Observation and Grade

One tissue sample from each stomach was processed and fixed in 10% formalin, and then embedded in paraffin. The embedded samples were cut into sections and stained with hematoxylin and eosin reagent for morphological observation and an evaluation of *H. pylori-*associated gastritis. Gastric tissue pathological scores were blindly assessed according to published methods [44]. HE-stained sections were observed and scored according to the following criteria: Grade 0, none; Grade 1, a few leukocytes distributed in the deep sites of mucosa; Grade 2, a moderate number of leukocytes distributed in mucosa from deep to mid sites and occasional neutrophils scattered in the gastric glands; Grade 3, dense infiltrates scattered in mucosa from the deep to mid sites; and Grade 4, diffuse and dense infiltrates scattered in the lamina propria and even into the submucosa.

### 3.6. Determination of LPO Level and MPO Activity

Stomach lipid peroxidation was measured based on the reaction between malondialdehyde generated by the peroxidation of tissue cell lipids and thiobarbituric acid (TBA) using the method described by Ohkawa et al [45]. Briefly, the stomach tissues were cut into pieces and homogenized with lysis buffer, and homogenate was centrifuged at 2000× *g* for 5 min to collect supernatant and remove debris. Then, 200 μL of a detection reagent containing 0.38% TBA, 15% trichloroacetic acid and 0.25 M HCl was added to 100 μL supernatant, and the mixture was incubated in boiling water for 15 min. Then cooling to room temperature, in order to collect the supernatant, the reaction sample was centrifuged at 1000× *g* for 10 min, the absorbance of supernatant was measured at 532 nm. The level of lipid peroxidation (LPO) was expressed as pg/mg protein. MPO activity was determined according to the method described by Krawisz *et al.* [45]. Each 200 mg sample was cut into pieces in 1 mL of precooling hexadecyltrimethylammonium bromide (HTAB) buffer (containing 0.5% HTAB in 50 mM phosphate buffer, pH 6.0) on ice, transferred to a 2 mL centrifuge tube, and homogenized with an electric tissue homogenizer three times on ice. After collecting the homogenate, the homogenizer was carefully rinsed twice with 1 mL of precooling HTAB buffer. The collected homogenates and washes were mixed and sonicated on ice for 10 s, repeatedly freeze-thawed three times at −80 °C, and then centrifuged at 1500× *g* for 15 min at 4 °C. The supernatant was collected and assessed MPO activity. MPO activity was detected by adding 0.1 mL of the supernatant to 2.9 mL of reaction reagents (50 mM phosphate buffer, pH 6.0, containing 0.0005% hydrogen peroxide and 0.167 mg/mL *O*-dianisidine hydrochloride). The absorbance values were measured at 460 nm with a UV-visible spectrophotometer. One unit of MPO activity is defined as the amount needed to degrade 1 pmol of peroxide per minute at 25 °C. Above protein concentration was measured using a BCA protein assay kit which was obtained from Sangon Biotech Co., Ltd., (Shanghai, China). The operation was done according to the manufacturer’s protocol. An aliquot of the protein was used to determine LPO level and MPO activity.

### 3.7. Quantitative RT-PCR to Assess the Expression Level of iNOS and IL-8 mRNA

The method employed was as described previously in this study [46]. Briefly, total RNA extraction was that tissues were homogenized in 1 mL TRIZOL Reagent (Sangon Biotech Co., Ltd., Shanghai, China) then followed with chloroform extraction and isopropanol precipitation to obtain RNA. An analysis of relative mRNA levels to the endo-reference gene β-actin was performed using a One Step AMV RT-PCR Kit (Sangon Biotech Co., Ltd., Shanghai, China). The operation was done according to the manufacturer’s instructions. Primers specific to the endo-reference gene β-actin (Genbank: NM_007393) and target genes IL-8 (Genbank: NM_011339) and iNOS (Genbank: NM_010927) were synthesized by Sangon Biotech Co., Ltd., Shanghai, China. The primers used are provided in Table 1. All PCR reactions included 30 cycles of 5 min at 95 °C for reverse transcription, 40 s at 94 °C for degeneration, 40 s at 60 °C for annealing, and 60 s at 72 °C for elongation. Products were electrophoresed on a 10 g/L agarose gel and were observed and photographed in a BioRad gel image analysis system. The relative IL-8 and iNOS mRNA levels were normalized to the endo-reference gene β-actin. The values for the endo-reference gene β-actin were set at 1 in each animal. The experiment was repeated in triplicate.

**Table 1:** RT-PCR Primers.

Gene	Primer Sequence	Base Pair (bp)
iNOS	Forward-5′-ACAATACAAGATGACCCTA-3′	357
	Reverse-5′-CAGATGTTCCTCTATTTTT-3′	
IL-8	Forward-5′-ATGGCTGCTCAAGGCTGGTCC-3′	389
	Reverse-5′-ATTCTCTTGTTCTCAGGTC-3′	
β-Actin	Forward-5′-ATGGTGGGAATGGGTCAGA-3′	391
	Reverse-5′-CGTGAGGGAGAGCATAGCC-3′	

### 3.8. ELISA Assay for IL-8

The expression level of IL-8 in gastric mucosal tissues was determined using an enzyme-linked immunosorbent assay (ELISA). A mouse IL-8 assay kit was purchased from Wuhan Boster Bio-engineering limited company, (Wuhan, China). The operation was done according to the manufacturer’s instructions. The experiment was repeated in triplicate.

### 3.9. Western Blot Analyses for the Levels of iNOS, HO-1, Nrf2 and IκBα, the Degree of IκBα Phosphorylated

Total cell proteins were extracted and prepared from gastric mucosa. The amount of protein was measured using a BCA protein assay kit which was obtained from Sangon Biotech Co., Ltd., (Shanghai, China). The operation was done according to the manufacturer’s protocol. An aliquot protein of each sample was used in this study. The samples of protein were separated by SDS-polyacrylamide gel electrophoresis, then transferred onto PVDF membranes by electroblotting. After blocking using PBS containing 5% nonfat dry milk for 2 h at 37 °C, the membranes were incubated for 2 h at 37 °C with rabbit anti-iNOS (Wuhan Boster Bio-engineering Limited, Co., Wuhan, China), anti-phospho-IκBα, anti-IκBα, anti-HO-1, anti-Nrf2 and anti-actin antibodies (BBI Life Science, Sangon Biotech Co., Ltd., Shanghai, China). The levels of above protein expression were detected using anti-rabbit secondary antibody conjugated to horseradish peroxidase, followed to expose and develop by enhanced chemiluminescence (BBI Life Science, Sangon Biotech Co., Ltd., Shanghai, China). Actin was used as an internal control, and an optical density variation of at least 5% was required for differences between groups and deemed to be significant.

### 3.10. Statistical Analysis

Statistical analyses were carried out using SPSS software (version 11.5, IBM Corp., Armonk, NY, USA), and the results were expressed as the mean ± standard deviation (SD). Comparisons between groups were analyzed by *post hoc* tests in the analysis of variance, and differences between the two groups were considered as to be statistically significant when *p* < 0.05.

## 4. Conclusions

In conclusion, the present work revealed that resveratrol possesses protective effects on *H. pylori* associated gastritis through exerting a significant against oxidative stress and anti-inflammatory effect by the way of suppressing the expression levels of IL-8 and iNOS, blocking the activation of NF-κB, and improving the expression levels of the Nrf2 and HO-1 in *H.pylori*-infected gastric mucosa. Resveratrol could be used as a food component to prevent damage from *H. pylori*-associated gastritis and reduce the incidence of gastric cancer in humans.
